# Activity and phosphatidylcholine transfer protein interactions of skeletal muscle thioesterase Them2 enable hepatic steatosis and insulin resistance

**DOI:** 10.1016/j.jbc.2024.107855

**Published:** 2024-10-05

**Authors:** Yang Xie, Xu Liu, Wenpeng Liu, Logan R. Carr, Luke P. Lee, Norihiro Imai, Eric A. Ortlund, David E. Cohen

**Affiliations:** 1 Division of Gastroenterology, Hepatology & Endoscopy, Brigham and Women’s Hospital, Harvard Medical School, Boston, Massachusetts, USA; 2 Department of Biochemistry, Emory University, Atlanta, Georgia, USA; 3 Division of Renal Medicine, Division of Engineering in Medicine, Brigham and Women’s Hospital, Harvard Medical School, Boston, Massachusetts, USA; 4 Department of Gastroenterology and Hepatology, Nagoya University Graduate School of Medicine, Aichi, Japan

**Keywords:** fatty acid metabolism, glucose metabolism, insulin resistance, lipid binding protein, hepatocyte, skeletal muscle metabolism

## Abstract

Thioesterase superfamily member 2 (Them2), a long-chain fatty acyl-CoA thioesterase that is highly expressed in oxidative tissues, interacts with phosphatidylcholine transfer protein (PC-TP) to regulate hepatic lipid and glucose metabolism and to suppress insulin signaling. High-fat diet–fed mice lacking Them2 globally or specifically in skeletal muscle, but not liver, exhibit reduced hepatic steatosis and insulin resistance. Here, we report that the capacity of Them2 in skeletal muscle to promote hepatic steatosis and insulin resistance depends on both its catalytic activity and interaction with PC-TP. Two residues of Them2 catalytic site were mutated (N50A/D65A) to produce the inactive enzyme while maintaining its homotetrameric structure and interaction with PC-TP. Restoration of skeletal muscle expression in *Them2*^*−/−*^ mice using recombinant adeno-associated virus revealed that WT, but not N50A/D65A Them2, promoted high-fat diet–induced weight gain and hepatic steatosis. This was accompanied by greater impairment of insulin sensitivity in WT than N50A/D65A Them2. Pharmacological inhibition or genetic ablation of PC-TP attenuated these effects. In reductionist experiments, conditioned medium collected from WT primary cultured myotubes promoted excess lipid accumulation in oleic acid–treated primary cultured hepatocytes relative to *Them2*^*−/−*^ myotubes, which was attributable to secreted extracellular vesicles. Reconstitution of Them2 expression in *Them2*^*−/−*^ myotubes affirmed the requirements for catalytic activity and PC–TP interactions for extracellular vesicles to promote lipid accumulation in hepatocytes. These studies provide valuable mechanistic insights, whereby Them2 in skeletal muscle promotes hepatic steatosis and establish both Them2 and PC-TP as attractive targets for managing metabolic dysfunction-associated steatotic liver disease.

Thioesterase superfamily member 2 (Them2, synonym: acyl-CoA thioesterase 13 [Acot 13]) is an Acot that catalyzes the hydrolysis of long-chain fatty acyl-CoA esters into free fatty acids and CoA (1, 2). Them2 is highly expressed in the liver and oxidative tissues, including skeletal and cardiac muscle (3). *Them2*^*−/−*^ mice are protected against high-fat diet (HFD)–induced obesity, hepatic steatosis, and insulin resistance (4, 5). Them2 interacts with phosphatidylcholine transfer protein (PC-TP, synonym StarD2) (3), a sensor of the fatty acyl composition of membrane PCs (6), at the mitochondrial membrane (1). Them2–PCTP interactions limit fatty acid oxidation (FAO) rates (7) and suppress insulin signaling (8). In the setting of overnutrition, the global absence of Them2 or PC-TP is associated with reduced hepatocellular endoplasmic reticulum stress, hepatic glucose production, and increased hepatic insulin sensitivity (5, 6, 9, 10). Notwithstanding these collective observations, mice with liver-specific disruption of Them2 expression (*L-Them2*^*−/−*^) exhibited alterations in fatty acid and glycerolipid metabolism but were not protected against diet-induced obesity, hepatic steatosis, or glucose intolerance (11).

To ascertain whether extrahepatic Them2 expression contributes to susceptibility to HFD-induced weight gain, hepatic steatosis and insulin resistance, we generated mice lacking Them2 in tissues where it is most highly expressed: skeletal muscle, cardiac muscle, and adipose tissue (12). Only skeletal muscle-specific Them2 KO (S-*Them2*^−/−^) mice recapitulated the protection observed in *Them2*^−/−^ mice (12). This study further demonstrated that increased very low–density lipoprotein secretion in response to increased rates of FAO in skeletal muscle was a likely hepatoprotective mechanism against steatosis. The possibility of crosstalk between skeletal muscle and liver was evidenced by the capacity for conditioned media harvested from Them2-overexpressing C2C12 cells to suppress rates of FAO in Hepa1-6 cells.

The current study was designed to gain deeper insights into mechanisms by which Them2 in skeletal muscle controls hepatic lipid metabolism in response to HFD feeding by assessing whether these effects depend on both catalytic activity and interactions with PC-TP. Reconstituted skeletal muscle expression of WT Them2 and a catalytically inactive N50A/D65A Them2 mutant promoted weight gain, hepatic steatosis, and insulin resistance in *Them2*^*−/−*^ mice upon challenge with a HFD. Pharmacological inhibition of PC-TP or its genetic ablation selectively in the skeletal muscle of *Them2*^*−/−*^ mice eliminated the capacity of WT Them2 to induce metabolic dysfunction. Conditioned media harvested from myoblasts isolated from WT, but not *Them2*^*−/−*^ mice, enhanced fatty acid–induced lipid accumulation in hepatocytes cultured from WT mice. This effect was attributable to extracellular vesicles (EV), which when isolated, recapitulated the genotype-specific effects observed for the conditioned media. These findings provide mechanistic insights into crosstalk between skeletal muscle and the liver and suggest that the catalytic activity of Them2 and its interactions with PC-TP in skeletal muscle may represent therapeutic targets for managing metabolic dysfunction–associated steatotic liver disease (MASLD).

## Results

### Reconstitution of WT but not catalytically inactive Them2 in skeletal muscle promotes body weight gain in the setting of overnutrition

To determine the contribution of Them2 activity in skeletal muscle to HFD-induced hepatic steatosis, we generated a catalytically inactive mutant enzyme. Based on the Them2 crystal structure (Fig. S1*A*), the catalytically important residues Asn (N) 50 and Asp (D) 65 were each mutated to Ala (A) to create N50A/D65A Them2. Importantly, these point mutations did not disrupt the capacity of purified recombinant Them2 to form homotetramers (Fig. S1*B*), which constitutes the active enzyme configuration. Whereas we observed the anticipated kinetic constants for WT Them2 (K_m_ = 7.3 μM; V_max_ = 866 nM/min) using myristoyl-CoA as a substrate (1), there was a near absence of activity of N50A/D65A Them2 (Fig. S1*C*).

**Figure 1:**
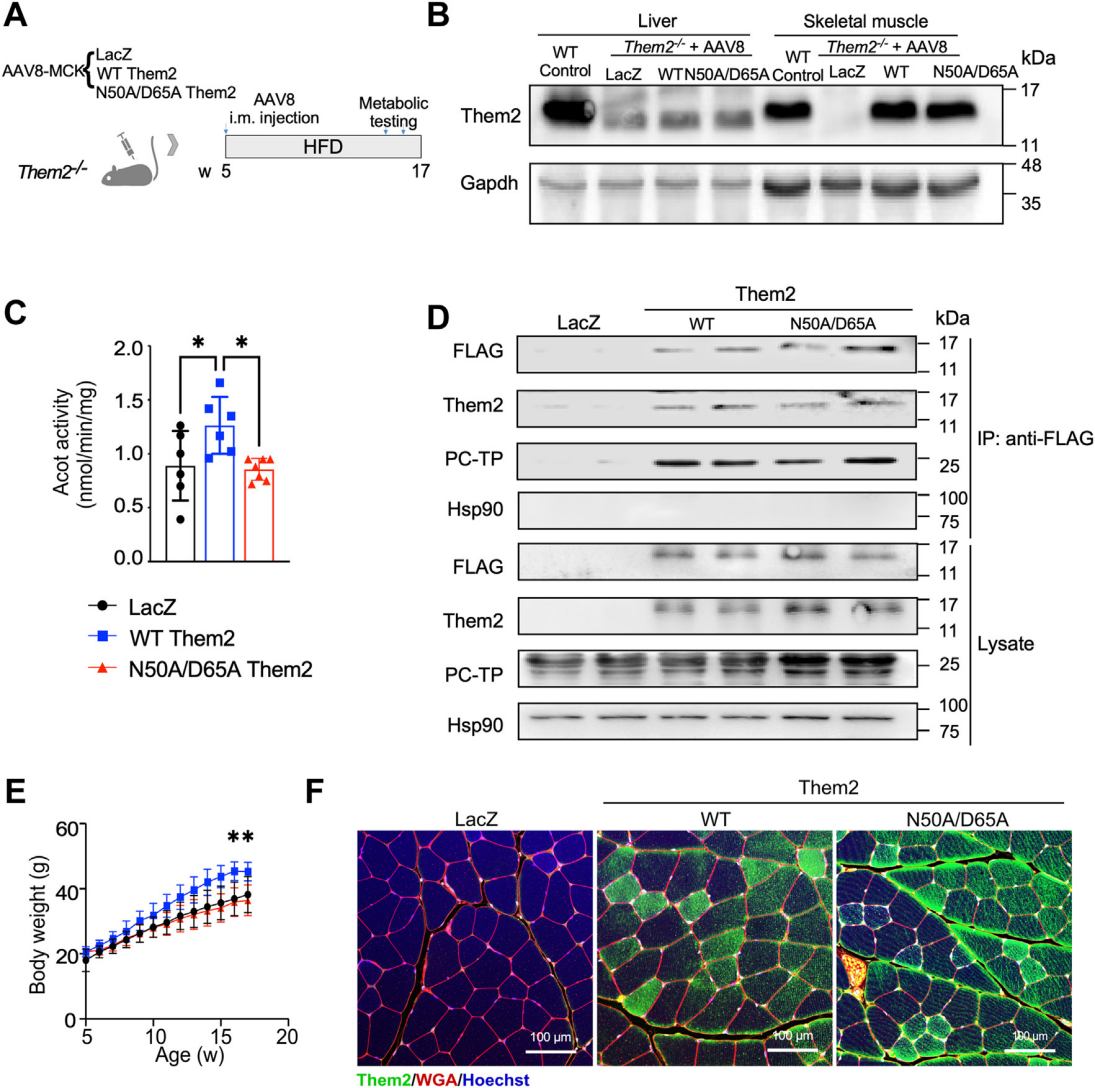
**Reconstitution of WT but not catalytically inactive Them2 in skeletal muscle promotes body weight gain in the setting of overnutrition.***A*, schematic representation for reconstitution of Them2 expression in HFD fed mice. Five-week-old *Them2*^*−/−*^ mice were i.m. injected with 4 × 10^11^ GC each of recombinant AAV8-MCK-WT Them2, AAV8-MCK-N50A/D65A Them2 or control AAV8-MCK-LacZ viruses. This was followed by 12 weeks of HFD feeding (from 5 to 17 weeks). *B*, immunoblot analyses showing AAV8-mediated Them2 expression in skeletal muscle, but not liver. To establish the dose of AAVs that generated comparable Them2 expression as WT control mice, protein expression in skeletal muscle was assessed 3 days following AAV8 administration by immunoblotting. *C*, using myristoyl-CoA as an exogenous substrate, V_max_ values of Acot activity were determined *ex vivo* 3 days following AAV8 administration for mitochondria isolated from gastrocnemius muscles. *D*, coimmunoprecipitation of PC-TP with FLAG-WT and FLAG-N50A/D65A Them2. Hsp90 served to control for unequal loading in the whole tissue lysate and as a negative control for immunoprecipitation by FLAG-WT and FLAG-N50A/D65A Them2. *E*, body weights of mice in response to HFD feeding. Data are mean ± SD; n = 5 to 7/group. Statistical analyses were conducted using one-way ANOVA with repeated measures; ∗*p* < 0.05, compared to control. *F*, immunofluorescence of WT or N50A/D65A Them2 in skeletal muscle (quadriceps) harvested following 12 weeks of HFD feeding, with LacZ serving as the control. Scale bars represent 100 μm. AAV, adeno-associated virus; Acot, acyl-CoA thioesterase; HFD, high-fat diet; i.m., intramuscularly; PC-TP, phosphatidylcholine transfer protein; Them2, Thioesterase superfamily member 2; WGA, wheat germ agglutinin.

Recombinant AAV8s were administered intramuscularly (i.m.) to achieve skeletal muscle reconstitution of Them2 (Fig. 1, *A* and *B*). By contrast, no AAV8-mediated expression of Them2 was observed in livers of *Them2*^*−/−*^ mice (Fig. 1*B*). Expression levels of WT Them2 and N50A/D65A Them2 were achieved in skeletal muscle of *Them2*^*−/−*^ mice that were similar to the endogenous expression of Them2 in skeletal muscle of WT control mice (Fig. 1*B*). Because Them2 is mitochondria-associated, we used mitochondria isolated from skeletal muscle to verify the Acot activity of WT Them2 and its absence in N50A/D65A Them2 reconstituted mice (Fig. 1*C*). Relative to the baseline activity of mitochondria from control mice transduced with LacZ (Fig. 1*C*) that reflects other mitochondrial Acot isoforms (13), mice expressing WT Them2 exhibited increased Acot activity, which was not observed for N50A/D65A Them2. Notwithstanding, PC-TP coimmunoprecipitated with both WT and N50A/D65A in skeletal muscle lysates (Fig. 1*D*), indicating that the mutations of the Them2 catalytic site were sufficient to abrogate Acot activity, but not interactions with PC-TP.

Compared with the LacZ control group, mice reconstituted with WT but not N50A/D65A Them2 exhibited excess body weight gain during 12 weeks of HFD feeding period (Fig. 1*E*). Upon sacrifice, the persistent expression of Them2 in skeletal muscle was validated by immunofluorescence microscopy (Fig. 1*F*). Collectively, these findings suggest that catalytic activity of Them2 in skeletal muscle contributes to excess weight gain in response to HFD feeding.

### Reconstitution of WT but not catalytically inactive Them2 in skeletal muscle promotes hepatic steatosis and myosteatosis

**Figure 2:**
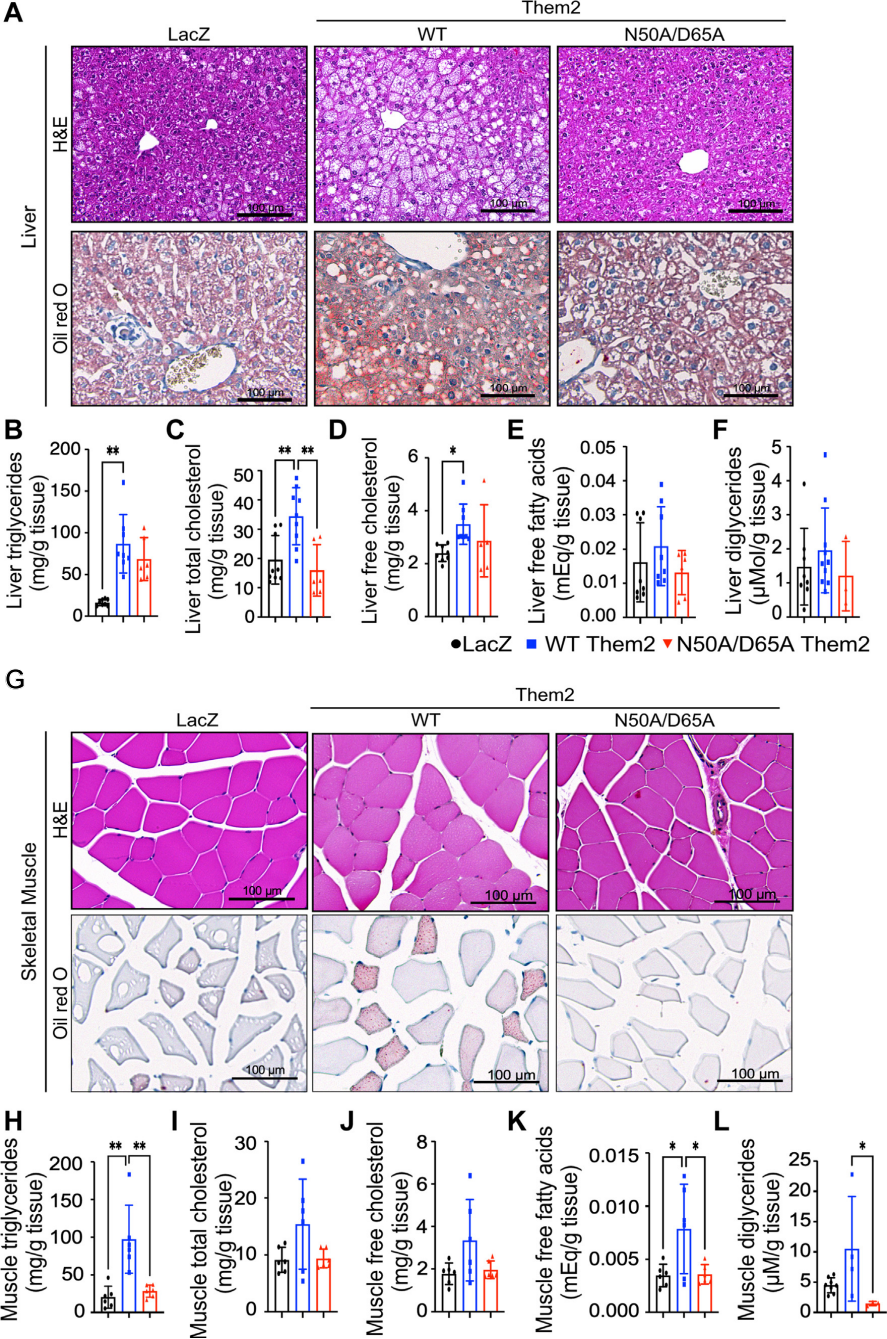
**Reconstitution of WT but not catalytically inactive Them2 in skeletal muscle promotes hepatic steatosis and myosteatosis.** Staining of (*A*) liver and (*G*) skeletal muscle using H&E (*top panels*) and oil red O (*bottom panels*). Scale bars represent 100 μm. *B*–*F*, hepatic and (*H*–*L*) skeletal muscle concentrations of (*B* and *H*) triglycerides, (*C* and *I*) total cholesterol, (*D* and *J*) free cholesterol, (*E* and *K*) free fatty acids, and (*F* and *L*) diglycerides. Data are mean ± SD; n = 3 to 8/group. Statistical analyses were conducted using one-way ANOVA with repeated measures; ∗*p* < 0.05 and ∗∗*p* < 0.01. Them2, Thioesterase superfamily member 2.

In line with the changes observed in body weight gain, reconstitution of WT but not N50A/D65A Them2 in the skeletal muscle promoted hepatic steatosis, as visualized by H&E staining and oil red O staining (Fig. 2*A*). These changes were associated with increased hepatic concentrations of triglycerides (Fig. 2*B*), total cholesterol (Fig. 2*C*), free cholesterol (Fig. 2*D*), free fatty acids (Fig. 2*E*), and diglycerides (Fig. 2*F*). These histopathological and biochemical changes in liver in HFD-fed mice are consistent with our previously published findings (5, 11, 12) and were largely mirrored in skeletal muscle, wherein reconstitution of *Them2*^*−/−*^ mice with WT Them2, but not N50A/D65A Them2 promoted myosteatosis. This was reflected by increased lipid droplet abundance in the skeletal muscle H&E and oil red O staining (Fig. 2*G*), along with increased intramuscular concentrations of triglycerides (Fig. 2*H*), total cholesterol (Fig. 2*I*), free cholesterol (Fig. 2*J*), free fatty acids (Fig. 2*K*), and diglycerides (Fig. 2*L*). These results reveal that the catalytic activity of Them2 in muscle contributed to diet-induced hepatic steatosis and myosteatosis.

### Reconstitution of WT but not catalytically inactive Them2 impairs the utilization of fatty acids and glucose and reduces insulin sensitivity

**Figure 3:**
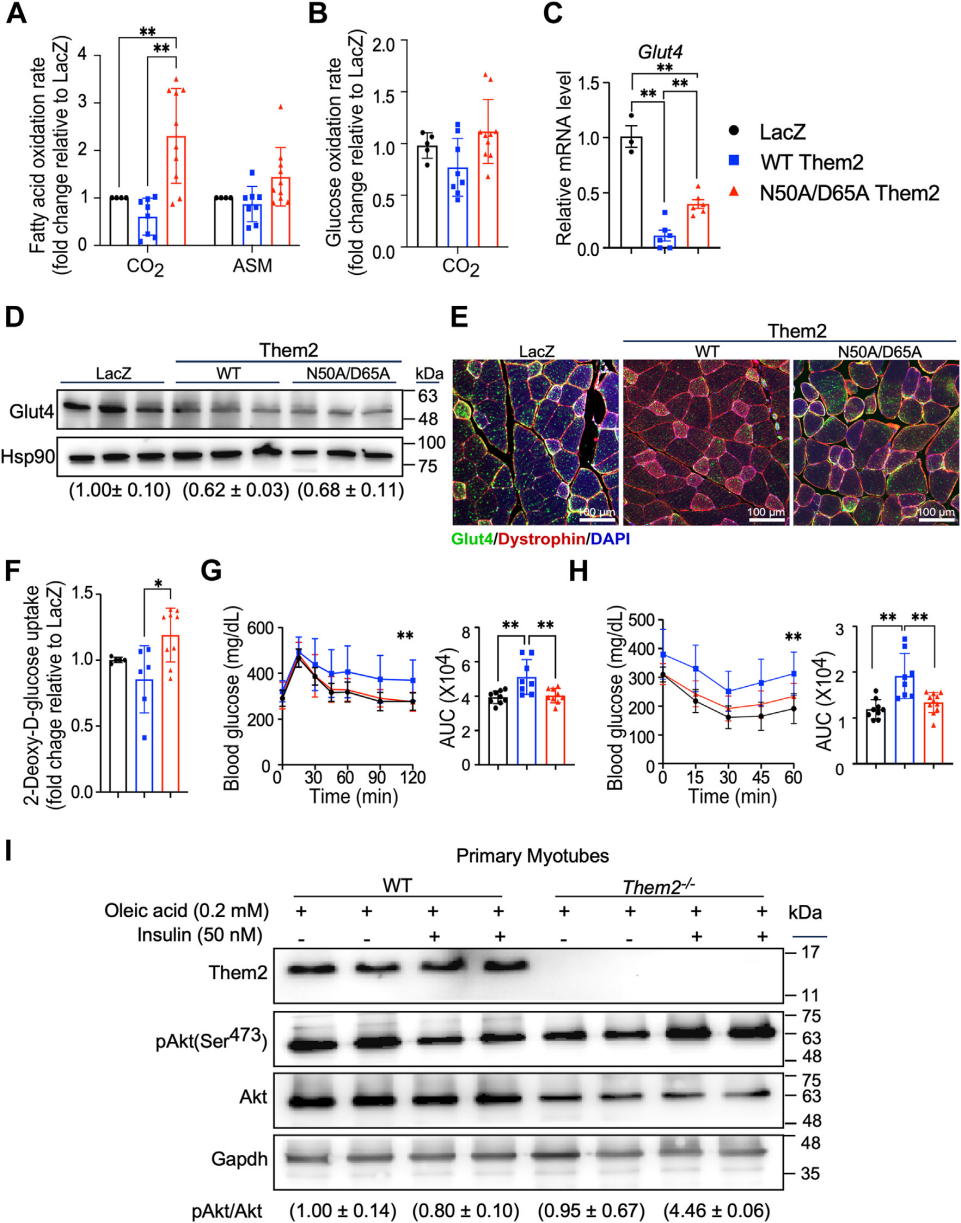
**Reconstitution of WT but not catalytically inactive Them2 impairs the utilization of fatty acids and glucose and reduces insulin sensitivity.***A*, rates of fatty acid oxidation in gastrocnemius as represented by [^14^C] CO_2_ and [^14^C] acid soluble metabolites (ASMs) produced from [^14^C] palmitic acid. [^14^C] ASM production accounted for > 98% of total [^14^C] palmitic acid degradation products. *B*, rates of glucose oxidation in gastrocnemius as represented by [^14^C] CO_2_ produced from [^14^C] glucose. Expression of Glut4 (*C*) mRNA, (*D*) protein (numbers in parentheses provide densitometric quantification (mean ± SD) of Glut4/HSP90 normalized to the LacZ control), as well as (*E*) plasma membrane localization. Scale bars represent 100 μm. *F*, rates of glucose uptake into soleus are represented accumulation of [^3^H]-2-DG in isolated soleus. *G*, oral glucose tolerance tests (*left*) and areas under the curve (AUCs) (*right*). *H*, insulin tolerance tests and areas under the curve (AUCs) (*right*). *I*, insulin-induced activation of Akt in OA (0.2 mM)-treated primary myotubes. The numbers in parentheses provide densitometric quantification (mean ± SD) of pAkt/Akt normalized to the WT control. These data represent two independent experiments. Data are mean ± SD; n = 6 to 9/group. Statistical analyses were conducted using one-way ANOVA (*B*–*C*, *F*–*H*) and two-way ANOVA (*A*) with repeated measures; ∗*p* < 0.05, ∗∗*p* < 0.01, compared to control. DG, deoxyglucose; Them2, Thioesterase superfamily member 2.

We have demonstrated that Them2 limits FAO in skeletal muscle (12). Indeed, the reconstitution of WT Them2 expression in skeletal muscle reduced FAO rates, as reflected by the production of both ^14^C-CO_2_ and ^14^C-labeled acid soluble metabolites (ASMs) from ^14^C-palmitate (Fig. 3*A*). We further observed that WT Them2 reduced rates of glucose oxidation (Fig. 3*B*), along with a decrease in mRNA (Fig. 3*C*), protein (Fig. 3*D*), and cell surface localization (Fig. 3*E*) of Glut4, which is in keeping with our prior observation that Glut4 was found significantly upregulated in S-*Them2*^−/−^ mice (12). Direct measurement glucose uptake (Fig. 3*F*) further showed suppression by WT Them. These effects were either abrogated or blunted by N50A/D65A Them2.

We have extensively characterized the protection against insulin resistance that occurs in response to 12 weeks of HFD in both *Them2*^*−/−*^ and *S-Them2*^*−/−*^ mice (5, 12). In keeping with WT Them2-induced insulin resistance as a mechanism of impaired glucose uptake and utilization in *Them2*^*−/−*^ mice, tolerance tests to glucose (Fig. 3*G*) and insulin (Fig. 3*H*) also exhibit impaired glucose homeostasis in the WT Them2-reconstituted group. This is further supported by findings of increased insulin-stimulated Akt phosphorylation in myotubes isolated from *Them2*^*−/−*^ mice relative to WT controls. Activation of key insulin signaling molecules, including pAKT and AKT were determined by immunoblotting following acute administration of insulin to differentiated WT or *Them2*^*−/−*^ primary myotubes in oleic-acid (OA) supplemented in the cell culture media; ablation of Them2 increased muscular insulin sensitivity, as evidenced by an increased proportion pAkt relative to Akt (Fig. 3*I*). The changes in myosteatosis, muscular fatty acid and glucose utilization, and insulin sensitivity suggest a critical role of the catalytic activity of skeletal muscle Them2 metabolism.

### PC-TP enables Them2 to induce insulin resistance, hepatic steatosis and myosteatosis

PC-TP is a key interacting partner of Them2 (3), and Them2-PC-TP interactions regulate both cellular metabolism (7) and insulin signaling (9). To discern whether PC-TP plays a role in Them2-induced hepatic steatosis, we utilized two loss-of-function models; a PC-TP inhibitor (14) that disrupts interaction with Them2 (8) and a newly generated skeletal muscle-specific Them2/PC-TP double knockout (S-DKO) mice.

**Figure 4:**
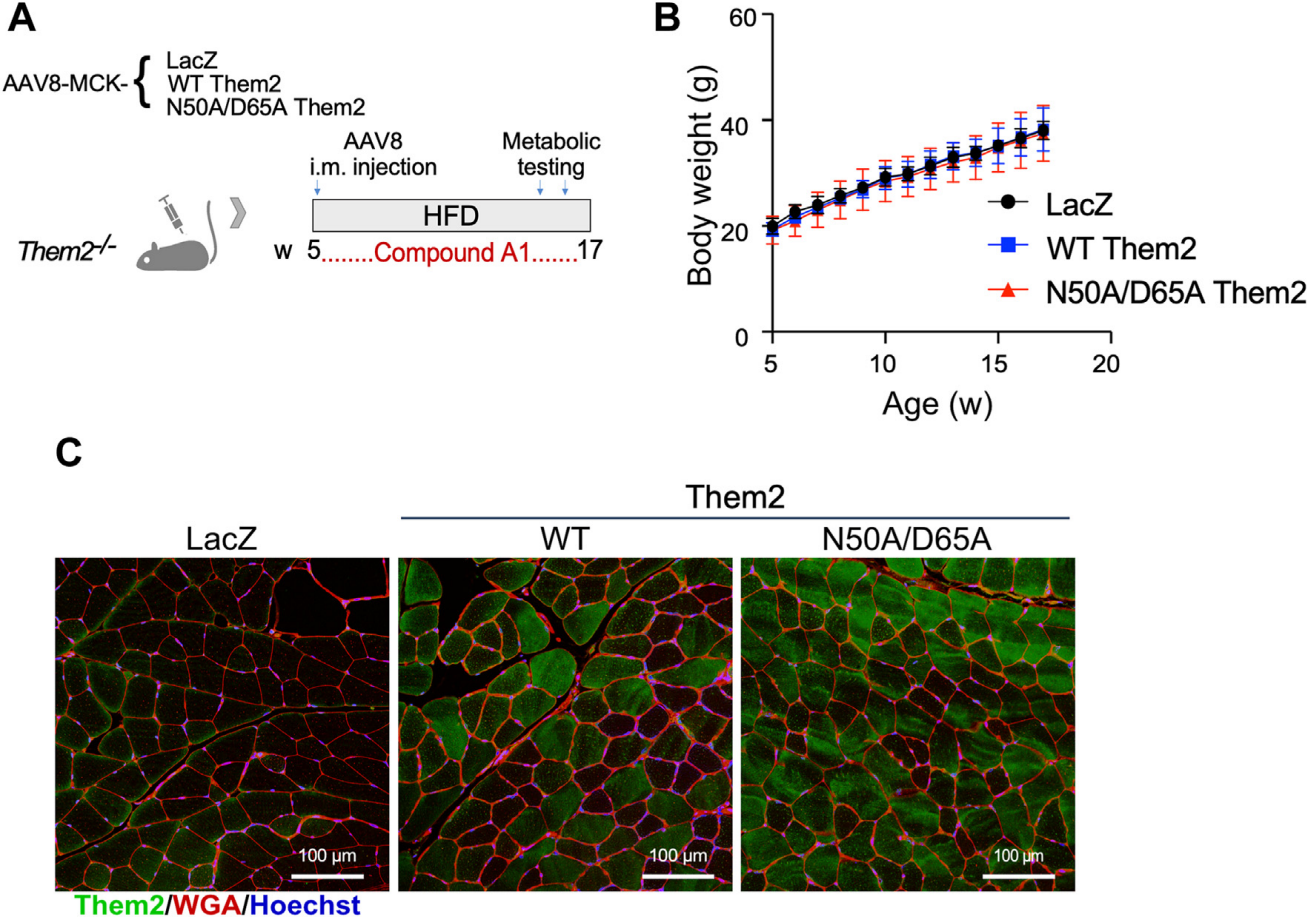
**PC-TP inhibition abrogates excess weight gain following reconstitution of WT Them2 in skeletal muscle.***A*, schematic of the experimental design to test the contribution of PC-TP to excess weight gain in response to HFD feeding promoted by reconstitution of WT Them2 in skeletal muscles of *Them2*^*−/−*^ mice. Mice were implanted with osmotic minipumps for the delivery of compound A1. This was followed by intramuscular injection of AAVs (4 × 10^11^ GC). Mice were then fed a HFD for 12 weeks. *B*, response of body weights to HFD feeding. *C*, immunofluorescence images of Them2 in quadriceps muscle tissue following 12 weeks of HFD feeding. Scale bars represent 100 μm. Data are mean ± SD; n = 5 to 6/group. AAV, adeno-associated virus; HFD, high-fat diet; i.m., intramuscular; PC-TP, phosphatidylcholine transfer protein; Them2, Thioesterase superfamily member 2.

**Figure 5:**
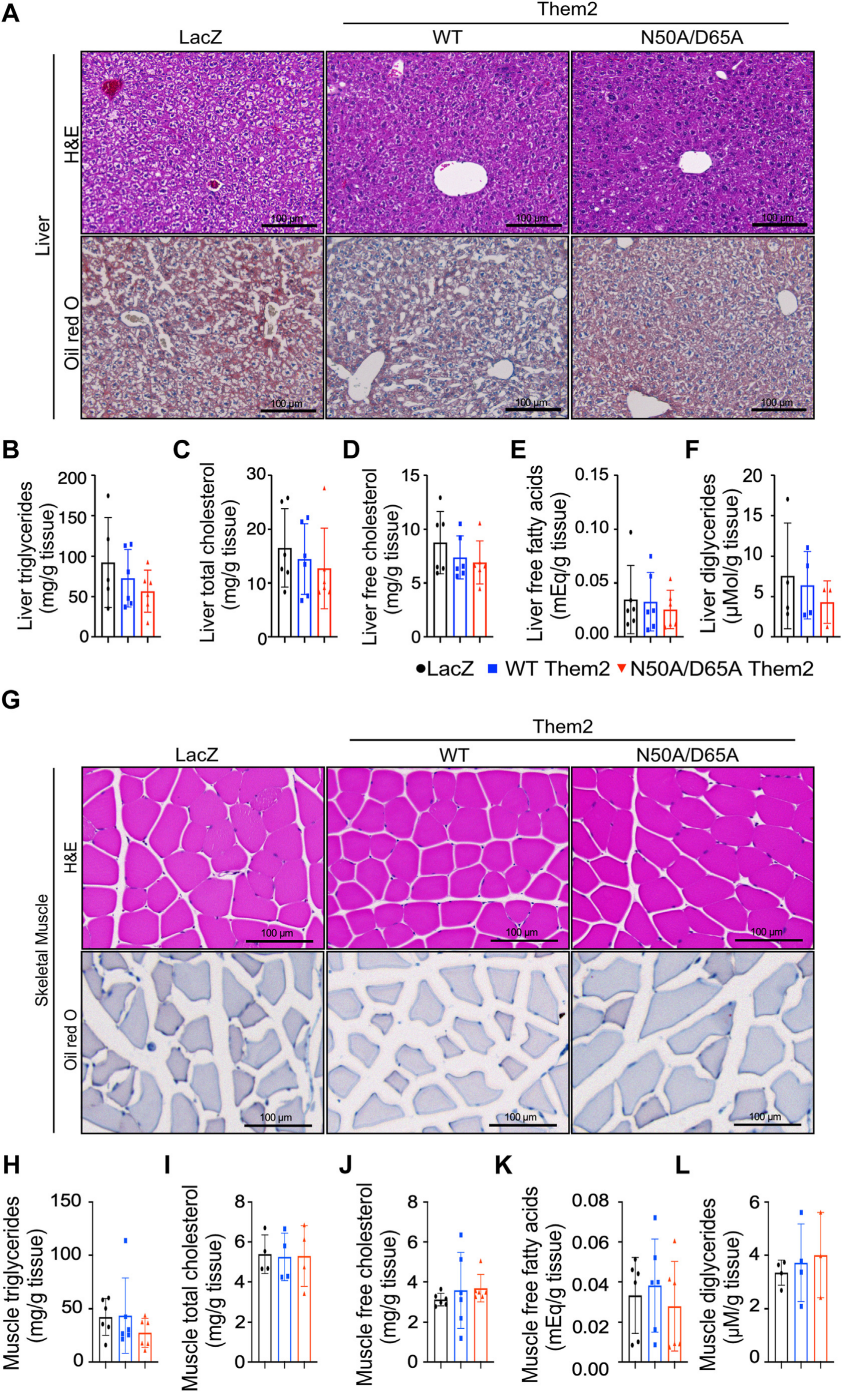
**PC-TP inhibition abrogates hepatic and myosteatosis following reconstitution of WT Them2 in skeletal muscle.** Staining of (*A*) liver and (*G*) skeletal muscle using H&E (*top panels*) and oil red O (*bottom panels*). Scale bars represent 100 μm. *B*-*F*, hepatic and (*H*–*L*) skeletal muscle concentrations of (*B* and *H*) triglycerides, (*C* and *I*) total cholesterol, (*D* and *J*) free cholesterol, (*E* and *K*) free fatty acids, and (*F* and *L*) diglycerides. Data are mean ± SD; n = 3 to 7/group. Statistical analyses were conducted using one-way ANOVA with repeated measures. PC-TP, phosphatidylcholine transfer protein; Them2, Thioesterase superfamily member 2.

As illustrated (Fig. 4*A*), *Them2*^*−/−*^ mice implanted with osmotic pumps were administered recombinant AAVs prior to a HFD feeding for 12 w, along with continuous compound A1 treatment at a dose that inactivates PC-TP *in vivo* (15). In contrast to *Them2*^*−/−*^ mice reconstituted with WT Them2 in the absence of PC-TP inhibition (Fig. 1*E*), no excess body weight gain was observed in mice treated with compound A1 (Fig. 4*B*). Following sacrifice, reconstitution of Them2 expression in skeletal muscle was affirmed by immunofluorescence staining (Fig. 4*C*). Compound A1 treatment abrogated the WT Them2-induced increase in hepatic steatosis, as assessed by histopathology (Fig. 5*A*) and hepatic concentrations of triglycerides (Fig. 5*B*), total cholesterol (Fig. 5*C*), free cholesterol (Fig. 5*D*), free fatty acids (Fig. 5*E*) and diglycerides (Fig. 5*F*). Compound A1 administration eliminated excess myosteatosis, as assessed by histopathology (Fig. 5*G*) and intramuscular lipid concentrations (Fig. 5, *H*–*L*).

**Figure 6:**
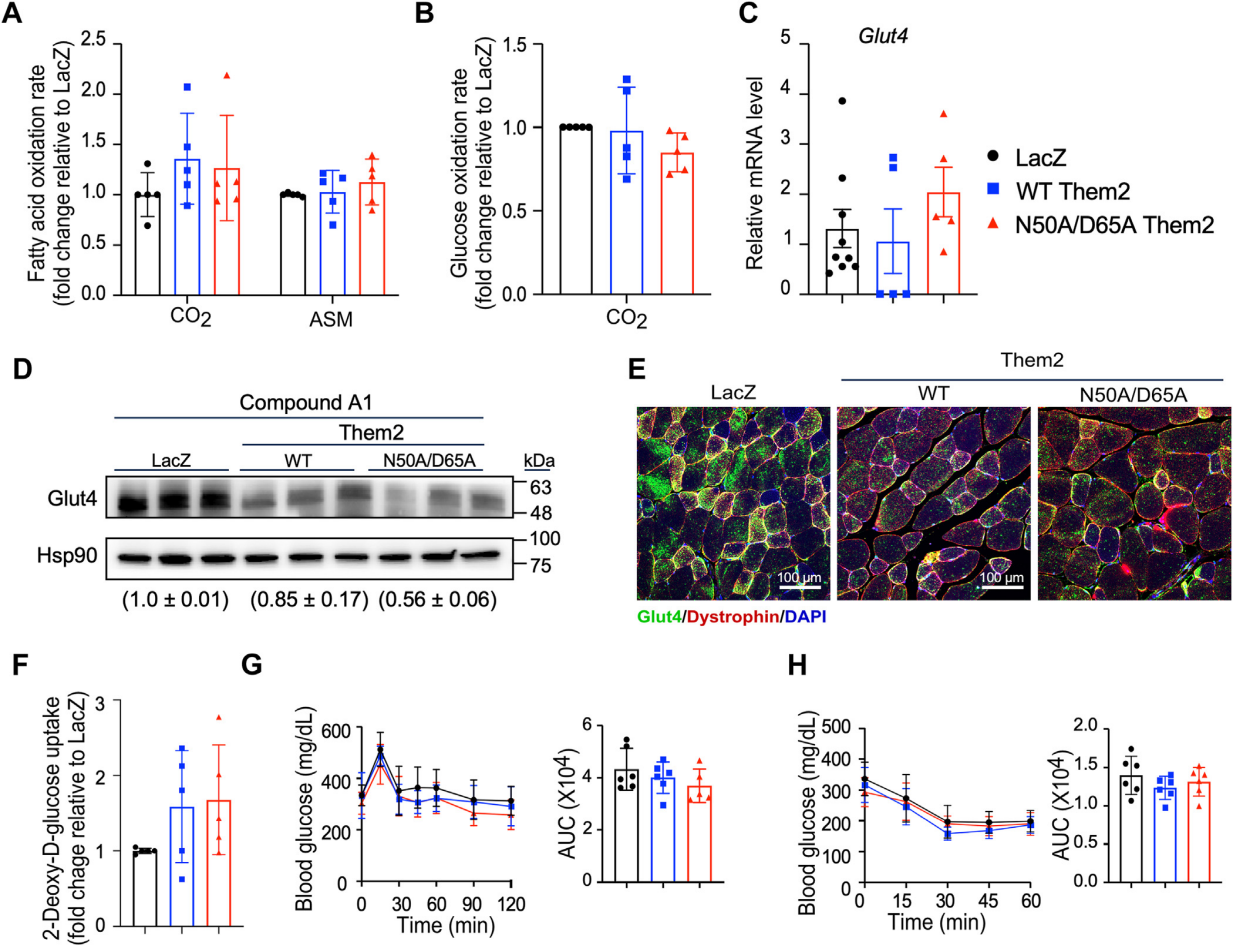
**PC-TP inhibition abrogates Them2-induced impairments in fatty acid and glucose utilization, as well as insulin sensitivity.***A*, rates of FAO in gastrocnemius as represented by [^14^C] acid soluble metabolites (ASMs) and [^14^C] CO_2_ produced from [^14^C] palmitic acid. [^14^C] ASM production accounted for > 98% of total [^14^C] palmitic acid degradation products. *B*, rates of glucose oxidation in gastrocnemius as represented by [^14^C] CO_2_ produced from [^14^C] glucose. Expression of Glut4 (*C*) mRNA, (*D*) protein (numbers in parentheses provide densitometric quantification (mean ± SEM) of Glut4/HSP90 normalized to the LacZ control), as well as (*E*) plasma membrane localization. Scale bars represent 100 μm. *F*, rates of glucose uptake into soleus as represented by accumulation of [^3^H]-2-DG. *G*, oral glucose tolerance tests (*left*) and areas under the curve (AUCs) (*right*). *H*, insulin tolerance tests and areas under the curve (AUCs) (*right*). Data are mean ± SD; n = 4 to 6/group. Statistical analyses were conducted using one-way ANOVA (*B*, *C*, *G*, and *H*) and two-way ANOVA (*A*) with repeated measures. DG, deoxyglucose; FAO, fatty acid oxidation; PC-TP, phosphatidylcholine transfer protein; Them2, Thioesterase superfamily member 2.

In response to compound A1 treatment, there were no differences in the oxidation rates of fatty acids (Fig. 6*A*) or glucose (Fig. 6*B*) among *Them2*^*−/−*^ mice reconstituted with WT Them2, N50A/D65AThem2, or LacZ. Relative to WT Them2, N50A/D65A Them2 did not alter changes in Glut4 expression (Fig. 6, *C* and *D*), plasma membrane localization (Fig. 6*E*) or glucose uptake (Fig. 6*F*). Similarly, the excess glucose intolerance (Fig. 3*G*) and insulin resistance (Fig. 3*H*) attributable to WT Them2 were not observed in mice treated with compound A1 (Fig. 6, *G* and *H*).

To further probe the contribution of PC-TP to the induction of hepatic steatosis by Them2 expression in skeletal muscle, we created S-DKO mice (Fig. S2, *A* and *B*). As observed for mice treated with compound A1 (Fig. 4*B*), no excess body weight gain was observed in mice reconstituted with WT Them2 relative to N50A/D65A Them2 or LacZ controls (Fig. S3*A*), as confirmed by immunofluorescence staining (Fig. S3*B*). Deletion of PC-TP in skeletal muscles abrogated the WT Them2-induced increase in hepatic steatosis, as assessed by histopathology (Fig. S3*C*) and hepatic concentrations of triglycerides (Fig. S3*D*), total cholesterol (Fig. S3*E*), free cholesterol (Fig. S3*F*), and free fatty acids (Fig. S3*G*). Deletion of PC-TP also eliminated excess myosteatosis, as assessed by histopathology (Fig. S3*H*) and intramuscular concentrations of triglycerides (Fig. S3*I*), total cholesterol (Fig. S3*J*), free cholesterol (Fig. S3*K*), and free fatty acids (Fig. S3*L*). Relative to the WT Them2, N50A/D65AThem2 did alter the plasma membrane localization of Glut4 (Fig. S3*M*). Tolerance tests to glucose (Fig. S3*N*) and insulin (Fig. S2*O*) were similarly unchanged.

### Myotube-derived extracellular vesicles promote fatty acid–induced lipid accumulation in primary hepatocytes

**Figure 7:**
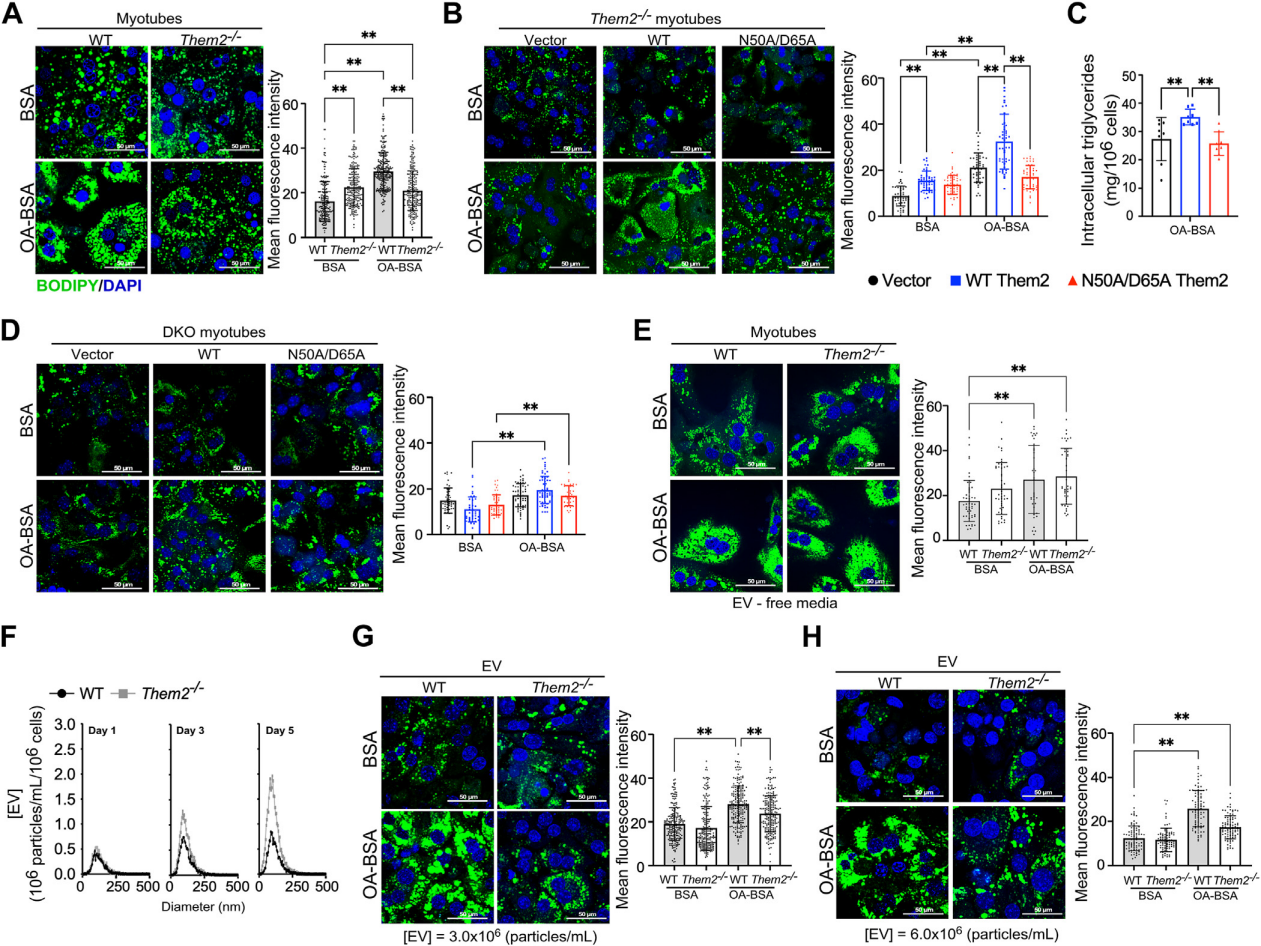
**Myotube-derived extracellular vesicles promote fatty acid–induced lipid accumulation in primary hepatocytes.***A*, representative images (*left*) and quantification (*right*) of BODIPY-stained neutral lipids in primary hepatocytes from WT mice treated with conditioned media from WT or *Them2*^*−/−*^ primary myotubes. *B*, representative images (*left*) and quantification (*right*) of BODIPY-stained neutral lipids in primary hepatocytes from WT mice treated with conditioned media from *Them2*^*−/−*^ primary myotubes reconstituted by plasmid transfection with vector or Them2 (WT or N50A/D65A). *C*, intracellular concentrations of triglycerides. *D*, representative images (*left*) and quantification (*right*) of BODIPY staining of neutral lipids in primary hepatocytes from WT mice treated with conditioned media from DKO primary myotubes reconstituted by plasmid transfection with vector or Them2 (WT or N50A/D65A). *E*, representative images (*left*) and quantification (*right*) of BODIPY-stained neutral lipids in primary hepatocytes from WT mice treated with conditioned media from WT or *Them2*^*−/−*^ primary myotubes following removal of EV by ultracentrifugation at 100,000*g* for 4 h. *F*, representative image of NTA demonstrating concentrations and size distributions of myotube-derived EV as functions of the day following differentiation. *G*, representative images (*left*) and quantification (*right*) of BODIPY-stained neutral lipids in primary hepatocytes from WT mice treated with culture media supplemented with EV (3.0 E + 06 particles/ml) purified from conditioned media collected from WT or *Them2*^*−/−*^ primary myotubes. *H*, representative images (*left*) and quantification (*right*) of BODIPY-stained neutral lipids in primary hepatocytes from WT mice treated with culture media supplemented with EV (6.0 E + 06 particles/ml) from conditioned media collected from WT or *Them2*^*−/−*^ primary myotubes. Data are mean ± SD. Statistical analyses were conducted using one-way ANOVA (*C*) and two-way ANOVA (*A*–*B*, *D*–*E*, *G*–*H*) with repeated measures; ∗∗*p* < 0.01. Quantification of fluorescent signals was analyzed on nine independent fields in each group using ImageJ. Scale bars represent 50 μm. DKO, double knockout; EV, extracellular vesicle; NTA, nanoparticle tracking analysis; Them2, Thioesterase superfamily member 2.

We previously demonstrated that conditioned medium from C2C12 myotubes transduced to overexpress Them2-suppressed basal and maximal oxygen consumption rates in Hepa1-6 cells relative to conditioned media from C2C12 myotubes transduced with vector alone (12). In keeping with the possibility of direct crosstalk between skeletal muscle and liver, we observed that conditioned media from WT myotubes (5 days postdifferentiation) promotes OA-induced lipid accumulation in primary cultured hepatocytes, an effect that was mitigated when using conditioned media from *Them2*^*−/−*^ myotubes (Fig. 7*A*). When conditioned media was collected from *Them2*^*−/−*^ myotubes transfected with vector control, WT Them2, or N50A/D65A Them2 plasmids, WT Them2, but not N50A/D65A Them2 in skeletal muscle, promoted lipid accumulation in the primary hepatocytes (Fig. 7*B*). Quantification of the hepatocellular triglyceride concentrations confirmed our observations using BODIPY for lipid quantification (Fig. 7*C*). To establish a role for PC-TP, conditioned media were collected from myotubes in *Them2*^−/−^*/Pctp*^−/−^ DKO mice following transfection with vector control, WT Them2 or N50A/D65A Them2 plasmids were exposed to primary cultured hepatocytes (Fig. 7*D*). Comparing of the lipid accumulation in response to OA with vector control and N50A/D65A Them2 groups, the increased lipid accumulation in response to OA (Fig. 7*B*) was not enhanced by WT Them2 (Fig. 7*D*), suggesting a cell autonomous requirement for PC-TP expression.

To gain additional mechanistic insights, we removed EV from conditioned media from WT and *Them2*^*−/−*^ myotubes (Fig. 7*E*). This abolished the inhibition of lipid accumulation in the hepatocytes by *Them2*^*−/−*^ conditioned media, discounting the likelihood that reduced lipid accumulation in the presence of conditioned media from *Them2*^*−/−*^ myotubes was attributable to the absence of a prosteatotic soluble factor, in favor of the presence of protective EV. To test this possibility, we isolated and characterized EV from the conditioned media. EV analyzed by nanoparticle tracking analysis showed that *Them2*^*−/−*^ myotubes produced and released more EV of all sizes than WT myotubes. This increase in EV release was observed to be time-dependent, with notably higher EV secretion from *Them2*^*−/−*^ myotubes on day 3 and day 5 postdifferentiation (Fig. 7*F*), suggesting Them2 expression suppresses EV production by myotubes. When equal concentrations of EV, 3.0 E + 6 particles/ml (Fig. 7*G*), and 6.0 E + 6 particles/ml (Fig. 7*H*) collected on day 5 postdifferentiation were used to supplement hepatocyte media, lipid accumulation was attenuated for EV from *Them2*^*−/−*^ relative to WT myotubes in response to OA treatment.

## Discussion

This study provides key mechanistic insights into the regulation of skeletal muscle metabolism by Them2, as well as potential crosstalk between skeletal muscle and the liver. We demonstrate that both the catalytic activity of Them2 and its interactions with PC-TP are required for Them2 to promote hepatic steatosis and insulin resistance in HFD-fed mice. Reductionist experiments reveal that the release of EV from primary cultured skeletal myocytes is regulated by Them2 and that the compositions of skeletal muscle–derived EV modulate the accumulation of triglycerides in primary cultured hepatocytes.

Our study was motivated by prior observations that the resistance to hepatic steatosis and insulin resistance observed in HFD-fed *Them2*^*−/−*^ mice (5, 9) was recapitulated by tissue-specific ablation of Them2 in skeletal muscle (12), but not in liver (11), cardiac muscle or adipose tissue (12). In support of the contribution of fatty acyl-CoA hydrolysis, our current *in vivo* studies revealed that catalytically inactive N50A/D65A Them2 expression in skeletal muscle failed to recapture excess myosteatosis and hepatic steatosis along with glucose intolerance, insulin resistance, and reduced muscle uptake of circulating glucose. In skeletal muscle of HFD-fed *S-Them2*^*−/−*^ mice (12), increased rates of FAO resulted in the accumulation of short-chain fatty acids, branched-chain amino acids, and selected pentose phosphate pathway metabolites that likely contributed to the improvement of skeletal muscle insulin sensitivity and glucose metabolism. Accordingly, reconstitution of *S-Them2*^*−/−*^ mice with WT Them2 suppressed rates of FAO, as well as glucose uptake and oxidation, an effect not observed N50A/D65A Them2 expression. Additionally, we observed in WT Them2—but not N50A/D65A Them2—reconstituted mice the accumulation of intramyocellular diglycerides, which contribute to muscle insulin resistance through activation of the novel protein kinase isoform PKCθ (16). This was associated with greater expression and plasma membrane translocation of Glut4 in skeletal muscle reconstituted with N50A/D65A Them2 than WT Them2.

We identified Them2 as a PC-TP–interacting protein, demonstrating that PC-TP stimulated the enzymatic activity of Them2 (3). Plausible metabolic contributions for a Them2–PC–TP complex in the regulation of hepatic glucose and lipid metabolism are supported by the overlapping phenotypes of *Them2*^*−/−*^ and *Pctp*^*−/−*^ mice (5, 9, 10, 15). Additional support has been provided by reductionist model systems both *in vitro* (1, 3) and in cells (7, 8, 9). Notwithstanding the robust support for the regulatory role of a Them2–PC–TP complex, evidence emerged for distinct roles for the complex in limiting fatty acid fluxes for oxidation by mitochondria (7, 9) or incorporation into glycerolipids (11), and suppressing insulin signaling (8, 15). In addition to evidence for the critical contribution of Them2 catalytic activity, our data support the contribution of PC–TP interactions. Both pharmacological disruption of Them2–PC–TP interactions with compound A1 and genetic ablation of skeletal muscle PC-TP abrogated WT Them2-induced hepatic steatosis and insulin resistance in the setting of over nutrition. Taken together, these findings demonstrate that both the enzymatic activity and interactions with PC-TP are integral to the capacity of Them2 to induce hepatic steatosis and insulin resistance.

We previously showed that C57BL/6J mice exhibit accelerated hepatic steatosis and insulin resistance in response to HFD feeding when mice exceed an average body weight threshold of 41.6 g (12). Above this threshold, there was a 4-fold increase in degree of hepatic steatosis per additional gram in body weight compared with mice with body weights of less than 41.6 g. An important observation was that selective ablation of Them2 expression limited weight gain so that mice uncommonly exceeded this threshold (*i.e.,* 20% after 12 weeks of HFD feeding *versus* 64% of controls). Our current findings confirm and extend this observation. Because they were bred to the C57BL/6J background, mice used in the current study could be placed in the context of our prior findings: Following 12 weeks of HFD-feeding, 89% (n = 8/9) of *Them2*^*−/−*^ mice reconstituted with WT Them2 exceeded the body weight threshold of 41.6 g (mean body weight ± SD, 44.8 ± 2.7 g) compared with only 11% (n = 1/9) of mice reconstituted with N50A/D65A Them2 (mean body weight ± SD, 37.1 ± 4.0 g) and 33% (n = 3/9) of LacZ control mice (mean body weight ± SD, 38.1 ± 5.9 g). These findings suggest that excess weight gain in response to Them2 activity in skeletal muscle contributes at least in part to enabling mice to exceed a critical weight threshold that accelerates the development hepatic steatosis.

An additional possibility for the contributions of Them2 to hepatic steatosis and insulin resistance is crosstalk between skeletal muscle and the liver. We previously showed that the absence of Them2 expression in skeletal muscle altered expression of secreted myokines, including interleukin-15, chemokine ligand 1, and *musclin*, suggesting a potential mechanism whereby skeletal muscle Them2 ablation may have limited the development of hepatic steatosis in response to HFD feeding (12). Additionally, conditioned media from C2C12 myotubes that overexpressed Them2 was capable of suppressing FAO in a Hepa1-6 hepatocyte cell line. Here, we confirm and extend our prior findings by using primary cultures cells. We show that activity was present in unfractionated conditioned media, but absent when the conditioned media was depleted of EV. Notwithstanding the previously observed changes in myokine gene expression, this indicates that soluble metabolites and secreted proteins were unlikely to be responsible. Moreover, activity was genotype-dependent, so that conditioned media from WT Them2-expressing primary myotubes promoted triglycerides accumulation in primary cultured hepatocytes, an effect not observed for N50A/D65A Them2. It was also PC-TP–dependent and localized to the EV fraction of the media. Our data also reveal that Them2 expression in skeletal muscle suppresses the production of EV *per se*.

Skeletal muscle produces EV that control metabolism in muscle, as well as other tissues, including the liver (17). EV derived from skeletal muscle, including those taken up by the liver, are significantly induced by exercise (18, 19). Isolated skeletal muscle–specific EV are enriched with the skeletal muscle-selective miR-206 (20), which can mitigate diet-induced hepatic steatosis and hyperglycemia (21). In addition to muscle-specific miRNAs (20), the skeletal muscle secretome comprises several hundred peptides (22, 23, 24), providing a conceptual basis for complex interorgan interactions with skeletal muscle. Future studies are expected to reveal the identities of EV components that are responsible for Them2-dependent crosstalk between skeletal muscle and the liver, as well as mechanisms by which Them2 controls rates of EV secretion.

This study has certain limitations. Some of the phenotypes of Them2-reconstituted mice did not fully recapitulate corresponding HFD-fed WT controls. There were no statistical differences between the WT and N50A/D65A Them2 reconstituted mice, with only a numerical downward trend for N50A/D65A Them2 (Fig. 2*B*). Similarly, there was only a downward trend for both fatty acid and glucose oxidation rates in WT Them2-reconstituted mice (Fig. 3, *A* and *B*). And although there were consistent trends, we neither observed significant differences in glucose uptake between the LacZ and WT groups nor between the LacZ and N50A/D65A Them2 reconstituted mice (Fig. 3*F*). One possibility for the lack of significant differences is that our experimental approach to reconstituting skeletal muscle Them2 expression using direct i.m. administration of adenoviral vectors did not fully restore its skeletal muscle expression throughout the body. Another possibility is that Them2 expression in liver may have been required to achieve a full phenotypic response of the liver to crosstalk from skeletal muscle. This is supported by the significant increase in intracellular triglycerides observed in WT primary hepatocytes treated with conditioned media from WT Them2-reconstituted primary myotubes (Fig. 7*C*). In this connection, we previously demonstrated cell autonomous regulatory effects on lipid and glucose metabolism of Them2 expression in hepatocytes (5, 7, 8, 9). Notwithstanding these caveats, our results taken together suggest that Them2 catalytic activity plays a key role in promoting steatosis of liver and muscle, as well as insulin resistance.

This study provides key mechanistic insights into the contribution of Them2 expression in skeletal muscle to the pathogenesis of hepatic steatosis and insulin resistance, by revealing the requirments for the Acot activity of Them2, as well as its interactions with PC-TP. Insulin resistance within skeletal muscle tissue contributes to excess weight gain, which together with EV-mediated crosstalk with the liver promotes hepatic steatosis. Whereas MASLD is the most prevalent chronic liver diseases and is rapidly increasing (25), current management options remain limited. A more comprehensive exploration of the impact of skeletal muscle Them2 on MASLD in mice would require the use of different diets (26), including those that promote progression of simple hepatic steatosis to include inflammation, hepatocyte apoptosis, and fibrosis. However, considering that hepatic steatosis and insulin resistance are central features of this common condition, our current findings implicate Them2 as a potentially attractive target in the medical management of MASLD.

## Experimental procedures

### Animals and diets

Mixed background *Them2*^−/−^ mice backcrossed 20 generations to C56BL/6J were as described (5). *Them2*^*−/−*^*/Pctp*^*−/−*^ DKO mice on the C56BL/6J background were generated by cross-breeding *Them2*^−/−^ and *Pctp*^*−/−*^ mice (5). Genotyping was performed by Transnetyx (Cordova) using real-time PCR. WT C56BL/6J mice (Stock #000664) were obtained from the Jackson Laboratory. Tissue-specific KO mice were created using a LoxP/Cre system (11, 12). C56BL/6J mice with *Them2* flanked by LoxP sites (*Them2*^*flox/flox*^) (11) were crossed with C56BL/6J *PC-TP*^*flox/flox*^ mice (27) to generate *Them2*^*flox/flox*^*/PC-TP*^*flox/flox*^ mice, which were crossed with mice expressing Cre recombinase driven by the Myf5 promoter (The Jackson Laboratory, Myf5-cre; Stock #007893) (12) to generate skeletal muscle deletion of both Them2 and PC-TP (S-DKO). These mice were viable and displayed no apparent developmental abnormalities. The presence of Cre allele was determined by PCR analysis using the primers specified by the Jackson Laboratory. Male mice were weaned at 4 weeks of age and fed chow (PicoLab Rodent Diet 20; LabDiet). Alternatively, 5-week-old male mice were fed a HFD (D12492: protein 20% kcal, fat 60% kcal, carbohydrate 20% kcal, energy density 5.21 kcal/g; Research Diets Inc.) for 12 weeks. In selected experiments PC-TP was inhibited using compound A1 (*4-[3-(2,4-dichlorobenzoyl)ureido]-N-(4,6-dimethylpyrimidin-2-yl)benzenesulfonamide*, LDN-193188) dissolved in 50% dimethylsulfoxide and 25% ethanol, which was administered for 12 weeks using implanted with ALZET osmotic minipumps Model 1002 (DURECT Corporation) at 3 mg/kg/d, a dose that we have demonstrated to inhibit PC-TP *in vivo* (15). Minipumps were replaced every 4 weeks. Mice were housed in a barrier facility on a 12 h light/dark cycle with free access to water and diet. Following 6 h fasting (8:00 AM to 2:00 PM), mice were euthanized, and plasma was collected by cardiac puncture. Tissues were harvested for immediate use or snap-frozen in liquid nitrogen and stored at −80 °C. The health status of mice was monitored by daily. Housing and husbandry were conducted in facilities with a sentinel colony health monitoring program and strict biosecurity measures to prevent, detect, and eradicate adventitious infections. Animal use and euthanasia protocols were approved by the Institutional Animal Care and Use Committee of Brigham and Women’s Hospital.

### Molecular cloning and mutagenesis

For bacterial expression, the full-length coding sequence of WT mouse Them2 was cloned into pET19b (Sigma). For mammalian expression, the same sequence inclusive of an N-terminal FLAG tag was cloned into a pcDNA3.1 vector (Thermo Fisher Scientific). Mutagenesis was performed using a QuikChange II XL Site-Directed Mutagenesis Kit (Agilent Technologies) and confirmed by nucleotide sequencing. The primer sequences for site-directed mutagenesis are provided in Table S1.

### Protein expression and purification

Recombinant proteins were expressed in *Escherichia coli* BL21 cells induced with 0.1 mM IPTG at 16 °C overnight. His-tagged Them2 proteins were purified using His-Trap affinity chromatography (GE Healthcare). Purified recombinant proteins (100 μM) were applied to an analytical Superdex 75 10/300 size-exclusion chromatography column at a flow rate of 0.5 ml/min. The elution profiles were established using UV absorbances at 280 nm.

### Adeno-associated virus-mediated Them2 expression in skeletal muscle

Skeletal muscle–specific expression of Them2 was achieved using adeno-associated virus serotype 8 (AAV8) (28) driven by the muscle creatine kinase (MCK) promoter (12). Briefly, expression of WT and mutant Them2 proteins was reconstituted in skeletal muscle of *Them2*^−/−^ and S-DKO mice was accomplished by direct i.m. administration of 4 × 10^11^ GC of AAV8 vectors. Recombinant AAV8 with transgenes driven by the Mck promoter included N-terminal FLAG (*i.e.,* DYKDDDDK)-tagged WT Them2 (AAV8-Mck-WT Them2) or FLAG-tagged N50A/D65A Them2 (AAV8-Mck-N50A/D65A Them2) packaged with AAV8-Mck-LacZ as a control (Charles River Laboratories, Vigene Biosciences). To ensure the absence of movement, mice were anesthetized (2% isoflurane). Shaved and sterilized hind limbs were injected with AAVs suspended in saline (12).

### Tolerance tests

Tolerance tests to glucose (GTT) and insulin (ITT) were as described with minor modifications (11, 12). Mice were fasted 6 h for GTTs and 2 h for ITTs. Glucose measurements were performed at baseline and at regular intervals using drops of blood obtained from tail tips (GE 100 Blood Glucose Monitor, General Electric). Glucose (1 g/kg body weight) was administered by orally gavage. Insulin (with 0.75 U/kg body weight) was administered by i.p. injection.

### Isolation and culture of primary hepatocytes and myoblasts

Mouse primary hepatocytes were isolated and cultured (8). Mice were anesthetized by i.p. injection of ketamine (200 mg/kg body weight; Webster Veterinary) plus xylazine (15 mg/kg body weight; Webster Veterinary). Inferior vena cavas were exposed, cannulated, and portal veins were severed in order to provide an exit route for the perfusate. Livers were perfused with 20 ml of Liver Perfusion Medium (Thermo Fisher Scientific) at 37 °C. This was followed by 30 ml of Liver Digest Medium (Thermo Fisher Scientific) also kept at 37 °C. Digested livers were removed, minced with a surgical blade in 10 ml of ice-cold Hepatocyte Wash Medium (Thermo Fisher Scientific), passed through a 70 μm filter (BD Biosciences), pelleted by gentle centrifugation (50*g* for 2 min) and washed 3x with 50 ml ice-cold hepatocyte wash medium. Cells were then resuspended in cold (4 °C) Williams E media (Thermo Fisher Scientific) containing 10% fetal bovine serum (FBS), 1 μM dexamethasone and 20 ng/ml epidermal growth factor. Viability was estimated by the trypan blue exclusion, and cells were plated on Primaria plates (BD Biosciences) at 80% confluence. Experiments were performed 48 h after plating. Transient transfections of plasmids were performed using Lipofectamine LTX (Thermo Fisher Scientific) according to the manufacturer’s protocol. Cells were transfected with plasmids (1 μg/ml) 24 h after plating at 50% confluence. Experiments were performed 48 h after plating.

Primary myoblasts were cultured and differentiated (29). Briefly, 60 mm culture dishes were treated with 2 ml of a coating solution (24 ml of Dulbecco's modified Eagle's medium (DMEM), 24 ml of HAMS F12, 1.7 ml collagen (A1048301, Thermo Fisher Scientific)) and 1 ml of Matrigel (356,234, Corning) at 4 °C for 1 h. Muscles tissues were dissected from a 4- to 8-week-old mice and gently rinsed in PBS containing 40 μg/ml gentamicin (G1397-10 Ml, Sigma). A sterile scalpel blade was used to gently slice muscles into small fragments (approximately 1–3 mm^3^), which were transferred onto the surface of a precoated 6 cm culture dish and gently overlaid with an additional 0.8 ml of plating media (12.5 ml of DMEM, 12.5 ml HAMS F12, 20 ml heat-inactivated FBS, and 5 ml amniotic fluid media supplement (12556023, Thermo Fisher Scientific). Culture dishes were then placed inside a homemade moist chamber (a 100 mm culture dish lined with 2–3 sheets of thick absorbent paper soaked with sterile water at the bottom) and incubated at 37 °C, 5% CO_2_ for 48 h, after which 2 ml of additional plating media was overlaid gently. Plates were maintained in the 37 °C incubator for an additional 3 days (5 days from original dissection) before harvesting the P0 myoblasts using trypsin. Myoblasts were seeded onto a precoated 6 cm dish with prewarmed myoblast media (17.5 ml DMEM, 17.5 ml HAMS F12, 10 ml FBS, and 5 ml amniotic fluid media supplement). To selectively harvest myoblasts, the heterogeneous P0 harvest (∼60% myoblasts) was subjected to two passages in PBS. P2 cells were cultured in precoated T75 flasks containing 10 ml of myoblast media. Transient transfection of plasmids (1 μg/ml) was performed using Lipofectamine LTX (Thermo Fisher Scientific) according to the manufacturer’s protocol using cells at 50% confluence. To differentiate primary myoblasts into myotubes, cells at 70 to 80% confluency were exposed to differentiation media (24 ml DMEM, 24 ml HAMS F12, and 1.5 ml heat-inactivated horse serum). Differentiation was marked by elongated, fused cells that spontaneously twitch. For conditioned media for further nano particle tracking analysis, primary myoblasts were cultured in myoblast media containing 20% exosome-depleted FBS (A2720803, Gibco). Conditioned media was collected on day 4 of differentiation. Insulin sensitivity was assessed on day 6 of differentiation following 8 h serum starvation. Cells were treated with insulin (50 nM) for 30 min.

### Isolation of mitochondria

Mitochondria were isolated from skeletal muscle essentially as described (30). Briefly, 100 mg of skeletal muscle tissue (dissected free from collagen and nerves) was minced into small pieces and suspended in 1 ml of isolation buffer (0.22 M mannitol, 0.007 M sucrose, 2 mM Tris, 1 mM EDTA, 20 mM Hepes, pH 7.2, and 0.4% albumin). The minced tissues were digested with 0.5 ml trypsin (0.3 mg/ml) for 5 min at room temperature, and then place immediately on ice. The digestion was stopped by adding 10 volumes of isolation buffer. The minced tissue was then homogenized, and centrifuged at 800*g* for 10 min. The supernatant centrifuged at 17,000*g* for 10 min, and the mitochondrial pellet was washed twice in 2.5 ml of isolation buffer and centrifuged at 17,000*g* for 10 min. The pellet was suspended in 50 μl of the same buffer for immunoblot analysis.

### Acot activity

Enzymatic activities of purified recombinant proteins were measured using myristoyl-CoA (Sigma) as the substrate, and 5,5′-dithiobis-(2-nitrobenzoic acid) (DTNB) to detect free CoA (1, 5). Enzymes were incubated in 30 mM Hepes buffer pH 7.5 containing 150 mM NaCl and 5% glycerol for 30 min at 37 °C. Myristoyl-CoA was added into 0.5 μM enzyme and 0.30 mM DTNB to initiate reactions. Values of absorbance at 412 nm were immediately recorded by temperature-controlled (37 °C) Synergy Neo 2.0 (BioTek) plate reader. Initial velocities (V_0_) were calculated by fitting the product formation during early time points using GraphPad Prism 9.0 (graphpad.com) for each substrate concentration. Values of initial rate of reaction (V_0_) were used to calculate values of K_m_ and V_max_ (1).

For an *ex vivo* essay of enzymatic activity, mitochondria were isolated and resuspended in lysis buffer (20 mM Tris, pH 8.0; 137 mM NaCl; 1 mM EDTA; 10% glycerol; and 0.5% Triton X-100), sonicated, and then incubated at 4 °C for 30 min. After centrifugation at 21,000*g* for 40 min at 4 °C, supernatants containing enzymes (50 μg protein) were combined with 50 μM myristoyl-CoA (Avanti Polar Lipids) dissolved in H_2_O. Reactions were initiated by adding prewarmed Acot activity assay buffer (50 mM KCl, 10 mM HEPES (pH 7.5), 0.3 mM DTNB) in each well of a 96-well plate to the final volumes of 200 μl. Recordings of absorbance values at 412 nm were commenced immediately recorded using a BioTek Cytation 5 Cell Imaging Multimode Reader (Agilent Technologies) and continued for 1 h at 37 °C.

### Tissue and cellular lipid concentrations

Lipids were extracted from frozen specimens or cultured cells using Folch’s method (11). Concentrations of triglycerides, cholesterol, free cholesterol, phospholipids, and free fatty acids were assayed enzymatically using reagent kits (FUJIFILM Wako Diagnostics).

### Rates of fatty acid oxidation

Rates of FAO were measured by degradation of 1-^14^C-palmitate (American Radiolabeled Chemicals) into ^14^C ASMs and ^14^C-labeled CO_2_ (11, 12). Briefly, gastrocnemius muscle strips were collected from following a 6 h fast. Muscle tissues were minced on ice and homogenized in a Dounce homogenizer followed by centrifugation for 10 min at 420*g*. Supernatants were transferred to microtubes containing 0.4 μCi ^14^C-palmitate mixed with 500 μM unlabeled palmitate conjugated with 0.7% fatty acid–free bovine serum albumin (BSA) and incubated at 37 °C for 30 min. ^14^C-labeled CO_2_ was captured onto filter papers soaked with 1 M NaOH, and ^14^C-labeled ASM were separated by the addition of 1 M perchloric acid. ^14^C-labeled CO_2_ in the filter paper and ^14^C-labeled ASM in the supernatant were each dissolved in Ecoscint H (National Diagnostics) and counted using an LS 6000IC liquid scintillation counter (Beckman Coulter).

### Rates of glucose oxidation

Rates of glucose oxidation in muscle tissues were measured by degradation of ^14^C(U)-glucose (PerkinElmer) into ^14^C-labeled CO_2_, by minor modification of a published protocol (31). Gastrocnemius muscle strips were collected, cut in half, weighed and preincubated for 30 min at 37 °C under gentle shaking (200 RPM) in 0.5 ml of modified Krebs–Henseleit buffer (KHB) containing 118 mM NaCl, 4.7 mM KCl, 2.5 mM CaCl_2_, 1.2 mM KH_2_PO_4_, 1.2 mM MgSO_4_, 15 mM NaHCO_3_, 1% fatty acid free BSA, 5 mM glucose, and 10 mM Hepes. Tissues were incubated for an additional 90 min at 37 °C with gentle shaking (200 RPM) in 0.5 ml KHB together with radiolabeled [1-^14^C]-glucose (0.5 μCi/ml). Reactions were terminated by addition of 100 μl 1 M perchloric acid, and the CO_2_ produced during the incubation was trapped onto the filter paper soaked with 1 M NaOH. ^14^C-labeled CO_2_ in the filter paper was dissolved in Ecoscint H and counted using an LS 6000IC liquid scintillation counter.

### Rates of glucose transport

Rates of glucose transport into isolated skeletal muscle were measured by the uptake of ^3^H-2-deoxyglucose (^3^H-2-DG; deoxy-D-glucose, 2-[1,2-^3^H(N)], PerkinElmer) into soleus muscle. The method to measure glucose transport was like that described previously with some modifications (31). Muscles were pre-incubated for 40 min at 30 °C in 2 ml of a modified KHB containing 118 mM NaCl, 4.7 mM KCl, 2.5 mM CaCl_2_, 1.2 mM KH_2_PO_4_, 1.2 mM MgSO_4_, 15 mM NaHCO_3_, 1% fatty acid free BSA, 5 mM glucose, and 10 mM Hepes. Thereafter, muscles were incubated an additional 20 min at 30 °C under gentle shaking (200 RPM) in 2 ml KHB plus ^3^H-2-DG (1.5 μCi/ml). Muscles were quickly removed, rinsed in sterile saline, and washed 3x in ice-cold KHB to remove extracellular ^3^H-2-DG. Muscles were then rinsed again in sterile saline, blotted with filter paper, weighed, and homogenized in 1 M KOH at 70 °C with periodic agitation for 15 min. Homogenates were centrifuged for 5 min at 10,000*g* at 4 °C. Supernatants were dissolved in Ecoscint H and were counted using an LS 6000IC liquid scintillation counter.

### Quantitative PCR

Total RNA was isolated from quadriceps muscle tissue (40 mg) using TRIzol (Thermo Fisher Scientific), treated with RNase-free DNase I, and reverse-transcribed into single-stranded complementary DNA. SYBR Green–based real-time PCR was performed with a QuantStudio 6 Flex Real-Time PCR System (Applied Biosystems). Data were normalized against the housekeeping gene β-actin. Relative gene expression was calculated using the ΔΔCT method, in which fold difference was calculated using according to 2−ΔΔCT. Primer sequences are provided in Table S2.

### Immunoblot analysis

Immunoblot analyses were performed as described (11, 12). Briefly, tissues or cells were homogenized using a Bead Ruptor 24 Elite bead mill homogenizer (Omni International) in radioimmunoprecipitation assay buffer containing complete Protease Inhibitor Cocktail and PhosphoSTOP Phosphate Inhibitor Cocktail Tablets (Roche). Protein concentrations were determined by using a bicinchoninic acid reagent (Thermo Fisher Scientific). Equal amounts of protein samples were separated by SDS-PAGE and transferred to nitrocellulose membranes for immunoblot analysis. Membranes were incubated with primary antibodies (Table S3) overnight and then probed with respective secondary antibodies (Dako-Agilent) for 1 h. Bands were developed using enhanced chemiluminescence (SuperSignal West DURA, Thermo Fisher Scientific) and imaged using a ChemiDoc XRS+ (Bio-Rad). Protein molecular weights were estimated using a Flash Protein Ladder (FPL-007, GelCompany) or a Thermo Scientific PageRuler Plus Prestained Protein Ladder (Thermo Fisher Scientific).

### Histopathology

For H&E staining, freshly harvested tissues were immersed in 10% neutralized formaldehyde for 24 h. Following paraffin embedding, sectioned tissues were stained by the Harvard Digestive Diseases Center Confocal Imaging Core B at Beth Israel Deaconess Medical Center. For oil red O staining, freshly harvested tissues were immersed in 10% neutralized formaldehyde overnight, followed by 30% sucrose overnight prior to embedding optical cutting temperature compound. Frozen-embedded tissues were then sectioned and stained with oil red O. H&E and oil red O–stained slides were visualized using a BioTek Cytation 5 Cell Imaging Multimode Reader (Agilent Technologies).

### Immunofluorescence microscopy

Tissue paraffin sections were incubated with a primary Glut4 antibody (ab33780, Abcam) and a primary dystrophin antibody (sc-73592, Santa Cruz) or wheat germ agglutinin (W6748, Invitrogen) were used as the surface markers of muscle fiber cells. 4′,6-Diamidino-2-phenylindole was used as a nuclear counterstain. For Them2 immunofluorescence staining, tissue paraffin sections were incubated with primary Them2 antibody (3). Hoechst was used as a nuclear counterstain. Paraffin-embedded tissues were sectioned and stained by the Harvard Digestive Diseases Center Confocal Imaging Core B at Beth Israel Deaconess Medical Center. Immunofluorescent images were visualized using a Zeiss LSM 880 Inverted Live-cell Laser Scanning Confocal Microscope (Zeiss).

### Isolation and characterization of EV

EV were isolated and characterized as described with modifications (32, 33). Primary myoblasts were cultured and differentiated (29). Exosome-depleted FBS (Gibco) was used in the cell culture media. Conditioned media was collected from T75 flasks of differentiated WT and *Them2*^*−/−*^ primary myoblasts, or *Them2*^*−/−*^ primary myoblasts transfected with plasmids. Samples of media were centrifuged at 2000*g* for 5 min at 4 °C to remove intact cells and debris. A portion of the conditioned media collected from myotubes was reserved for experiments using cultured hepatocytes. The remaining portion was used to prepared EV-free media by ultracentrifugation at 100,000*g* for 4 h or to purify EV purification using an EXODUS ultrafast-isolation system (32). Purified EV were characterized by nanoparticle tracking analysis by a ZetaView nanoparticle tracking analyzer (Analytik) at the Cell Function and Imaging Core of Boston Children’s Hospital.

### Staining and quantification of cellular neutral lipids

Primary hepatocytes (2 × 10^5^) from WT mice were seeded on 12-well culture plates fitted with collagen-coated No. 1 round cover glasses (Azer Scientific). After 24 h of incubation, the media was removed and replaced with conditioned media containing vehicle BSA or BSA-OA (OA 0.2 mM) for 24 h. Cells were washed twice with PBS and fixed with 4% paraformaldehyde in 4% sucrose dissolved in PBS for 20 min. Fixed cells were again washed twice with PBS and then stained with a PBS containing solution BODIPY 493/503 (1:1000, GC42959, GLPBIO) for 30 min at 37 °C (34). After washing twice with PBS, images were taken using a Zeiss LSM 880 Inverted Live-cell Laser Scanning Confocal Microscope (Carl Zeiss). ImageJ (imagej.net) software was used for the quantification of fluorescent signals by analyzing nine independent visual fields.

### Coimmunoprecipitation of Them2 and PC-TP

Interactions between FLAG-tagged Them2 proteins and PC-TP were assessed by immunoprecipitation. Magnetic agarose beads (Pierce Anti-DYKDDDDK (FLAG) Magnetic Agarose, Invitrogen) were equilibrated to room temperature. 450 μl lysis buffer (25 mM Tris–HCl, pH 7.4, containing 150 mM NaCl, 1 mM EDTA, 1% NP-40, and 5% glycerol) was added to 50 μl magnetic agarose beads and gently vortex mixed. The agarose beads were allowed to settle, and the supernatant was discarded. This wash was repeated twice for a total of 3x. Samples of gastrocnemius muscle tissues (400 μg) collected 7 days following i.m. injections of AAV8 constructs were homogenized in 300 μl lysis buffer using pestles (Fisherbrand RNase-Free Disposable Pellet Pestles, Thermo Fisher Scientific). The lysates were centrifuged at 13,000*g* for 20 min at 4 °C, and the supernatants were collected. The supernatant was added to the prewashed magnetic agarose and incubated at 37 °C with mixing for 40 min. The beads were collected using a magnetic stand and washed 3x with lysis buffer. Beads containing proteins were denatured by heating (5 min at 70 °C) in Laemmli buffer and then subjected to SDS-PAGE and immunoblot analysis.

### Statistical analyses

Statistical significance was adjudicated using two-tailed unpaired Student’s *t-*tests when two groups were compared. Multiple group comparisons were performed using one-way ANOVA when using one independent variable to compare the means of three or more groups of data or two-way ANOVA when using two independent variables to compare the means of three or more groups of data. Differences were considered significant for *p* < 0.05 (GraphPad Prism version 9.4.1, GraphPad Software).

## Data availability

All data are contained within this article.

## Supporting information

This article contains supporting information (3, 35).

## Conflict of interest

The authors declare that they have no conflicts of interest with the contents of this article.
