# Supplementary material for: Activity and phosphatidylcholine transfer protein interactions of skeletal muscle thioesterase Them2 enable hepatic steatosis and insulin resistance
Source: J Biol Chem. 2024 Oct 5;300(11):107855. doi: 10.1016/j.jbc.2024.107855 (PMC11570472; doi:10.1016/j.jbc.2024.107855)
Supplement: Supporting information [file mmc1.docx]

**Supporting Information**

**Activity and phosphatidylcholine transfer protein interactions of skeletal muscle thioesterase Them2 enable hepatic steatosis and insulin resistance**

Yang Xie^1^, Xu Liu^2^, Wenpeng Liu^3^, Logan R. Carr^1^, Luke P. Lee^3^, Norihiro Imai^4^, Eric A. Ortlund^2^, David E. Cohen^1,5^

^1^Division of Gastroenterology, Hepatology & Endoscopy, Brigham and Women’s Hospital, Harvard Medical School, Boston, Massachusetts, USA

^2^Department of Biochemistry, Emory University, Atlanta, Georgia, USA

^3^Division of Renal Medicine, Division of Engineering in Medicine, Brigham and Women’s Hospital, Harvard Medical School, Boston, Massachusetts, USA

^4^Department of Gastroenterology and Hepatology, Nagoya University Graduate School of Medicine, Aichi, Japan

^5^Corresponding author: Brigham and Women’s Hospital, 77 Avenue Louis Pasteur, Room 250A, Boston, MA 02115**,** USA. Tel: +1 (617) 525 1260. E-mail address: [dcohen@bwh.harvard.edu](mailto:dcohen@bwh.harvard.edu)

**Materials included:**

Supplemental figures 1-3

Supplemental tables 1-3


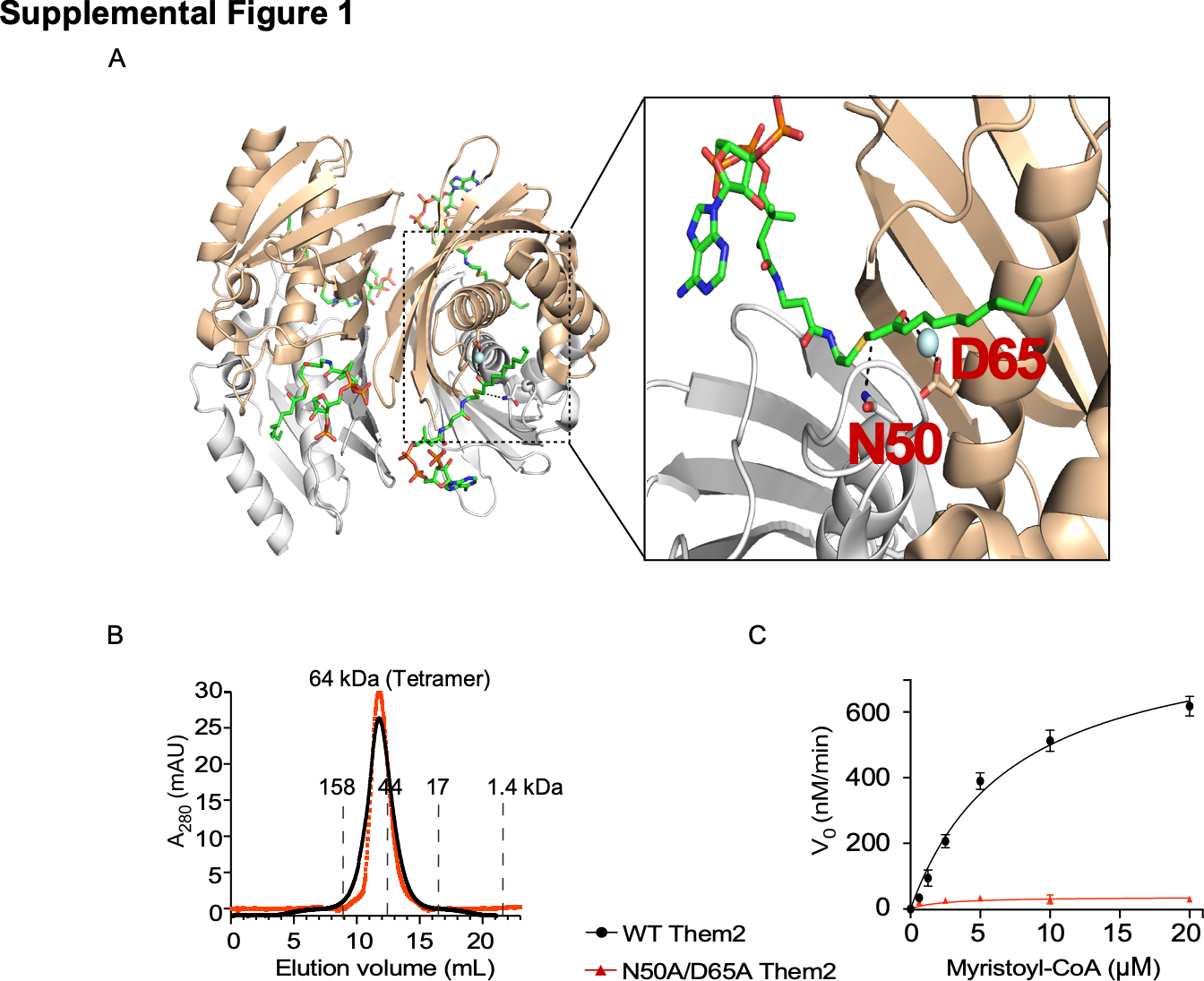


**Supplementary Figure 1. Point mutations in the catalytic site abrogate activity but not self-association of Them2.**

**(A)** Cartoon representation of the Them2 tetramer with bound C11-CoA molecules represented as sticks (PDB 3F5O), including a close-up view of the active site showing catalytically important residues Asn (N) 50 and Asp (D) 65. Subunits A and C are shown in white, with B and D in wheat. **(B)** Size exclusion chromatography of purified recombinant WT Them2 and N50A/D65A Them2. Relative to the protein standards indicated, WT Them2 (16 kDa) eluted at a volume that is indicative of a homotetramer (64 kDa). Them2 N50A/D65A eluted at same volume, implying that these mutations did not influence self-assembly. **(C)** Acot activity assay for purified recombinant proteins using myristol-CoA as the substrate.


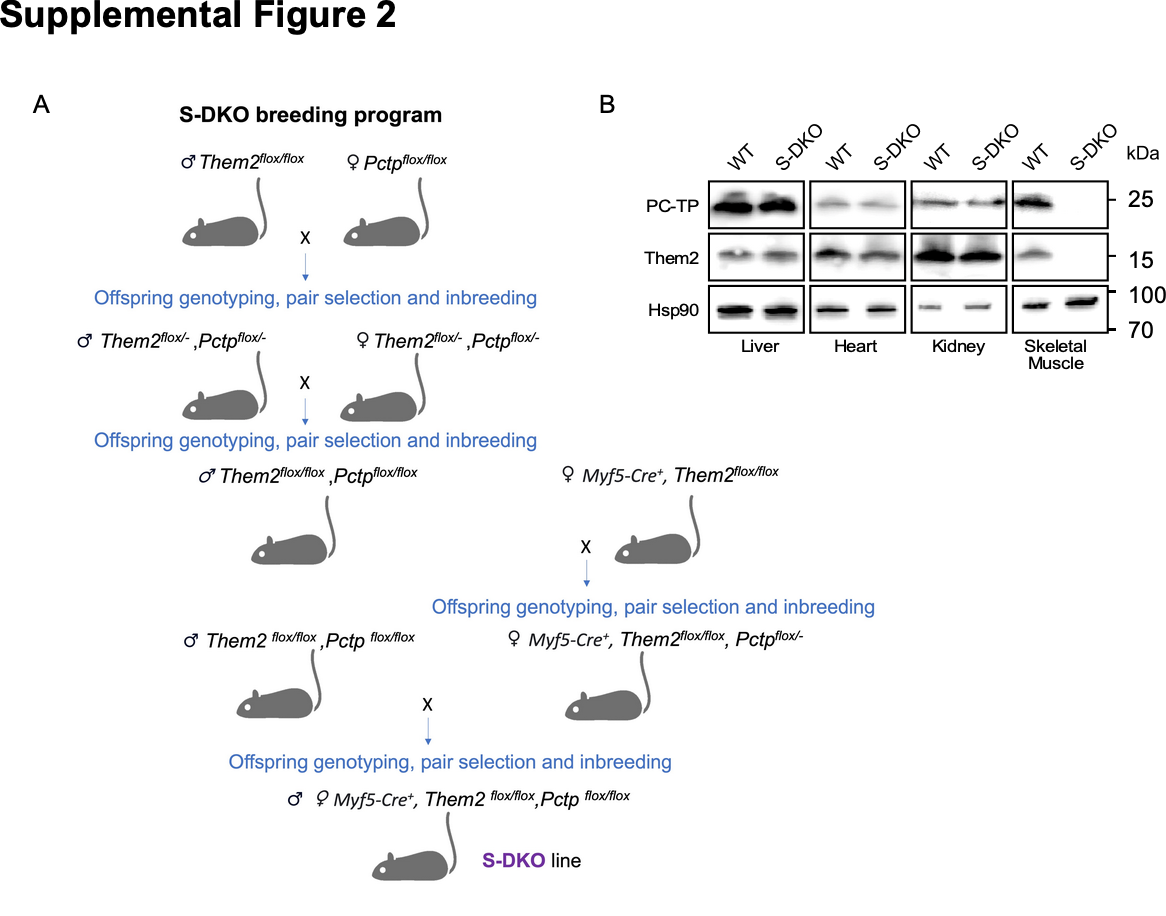


**Supplementary Figure 2. Generation of skeletal muscle-specific Them2/PC-TP double knockout (S-DKO) mice.**

**(A)** Schematic representation of the crossbreeding between *Them2^flox/flox^/Pctp^flox/flox^* transgenic mice (the 3^rd^ generation offspring of *Them2^flox/flox^*and *Pctp^flox/flox^*) and *Myf5-Cre^+^* mice to generate the *Myf5-Cre^+^*,*Them2^flox/flox^/Pctp^flox/flox^* mice. **(B)** Validation S-DKO mice by immunoblotting.


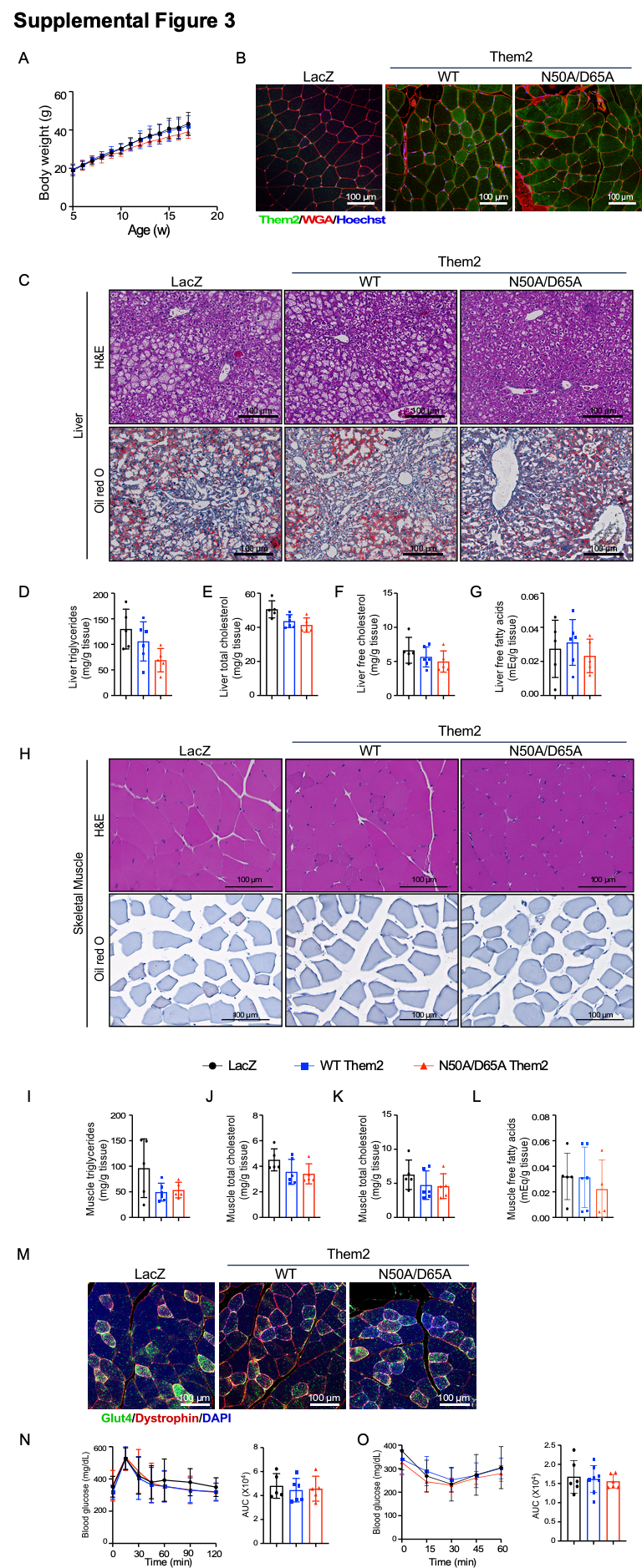


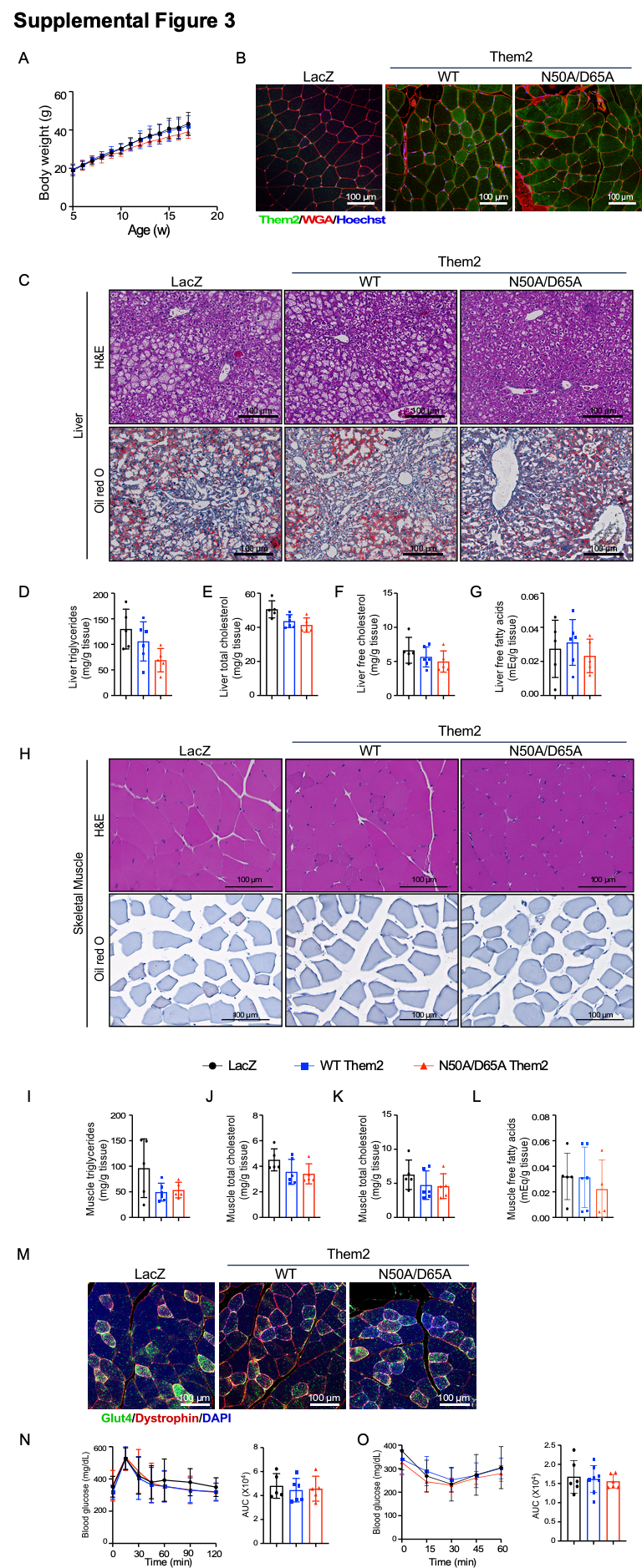


**Supplementary Figure 3. Ablation of Pctp in skeletal muscle abrogates Them2-induced impairment of glucose homeostasis, hepatic steatosis and myosteatosis.**

**(A)** Body weights of skeletal muscle WT Them2 - reconstituted - S-DKO mice and the LacZ control group. **(B)** The expression of WT Them2 and N50A/D65A Them2 in skeletal muscle measured by immunofluorescent staining. **(C,H)** Liver and skeletal muscle histology were examined using H&E and oil red O staining. Bars represent 100 μm. **(D-G)** Hepatic and **(I-L)** skeletal muscle concentrations of **(D,I)** triglycerides, **(E,J)** total cholesterol, **(F,K)** free cholesterol, and **(G,L)** free fatty acids. **(M)** Plasma membrane localization of Glut4. Bars represent 100 μm. **(N)** Oral glucose tolerance test (left) and area under the curve (AUC) (right). **(O)** Insulin tolerance test and area under the curve (AUC) (right). **(**Data are mean ± SD; n=3–6/group. Statistical analyses were conducted using one-way ANOVA with repeated measures; *p < 0.05, **p <0.01, compared to control.

**Supplemental Table 1. DNA oligos used in site-directed mutagenesis**

| Oligo name | Forward Primer (5’ – 3’) | Reverse primer (5’ - 3’) |
| --- | --- | --- |
| *mThem2 D65A* | ACAGCAACCTTAGTGGCCAGCATCTCGACCATG | CATGGTCGAGATGCTGGCCACTAAGGTTGCTGT |
| *mThem2*  *N50A* | GAAGAGCAGCATACTGCTAAACTGGGCACGCTC | GAGCGTGCCCAGTTTAGCAGTATGCTGCTCTTC |

**Supplemental Table 2. Mouse primers used in real-time PCR analysis**

| Gene | NCBI access number | Forward Primer (5’ – 3’) | Reverse primer (5’ - 3’) |
| --- | --- | --- | --- |
| *Glut4* | NM_001359114.1 | CATTTGGGGCCCTAGGTTGT | GGGTTTCTGCTCCCTATCCG |
| *Gapdh* | NM_008084.2 | TGTGTCCGTCGTGGATCTGA | TTGCTGTTGAAGTCGCAGGAG |

**Supplemental Table 3. Antibodies used in immunoblotting (WB/IF)**

| Antibody | Manufacturer | Catalog # |
| --- | --- | --- |
| Akt(pan) | Cell Signaling | 4691S |
| pAkt (S473) | Cell Signaling | 4060S |
| Distrophin | Millipore Sigma | MABT827 |
| Flag | Abcam | ab125243 |
| Glut4 | Abcam | ab33780 |
| Gapdh | Invitrogen | 2118 |
| Hsp90 | Santa Cruz | sc-13119 |
| PC-TP | Shoda *et al*., 2001^a^ |  |
| Them2 | Kanno *et al.*,2007^b^ |  |
| WGA | Invitrogen | W6748 |

*^a^*Shoda, J., Oda, K., Suzuki, H., Sugiyama, Y., Ito, K., Cohen, D. E. *et al.* (2001) Etiologic significance of defects in cholesterol, phospholipid, and bile acid metabolism in the liver of patients with intrahepatic calculi *Hepatology* **33**, 1194-1205

*^b^*Kanno, K., Wu, M. K., Agate, D. S., Fanelli, B. J., Wagle, N., Scapa, E. F. *et al.* (2007) Interacting proteins dictate function of the minimal START domain phosphatidylcholine transfer protein/StarD2 *J Biol Chem* **282**, 30728-30736
